# Immune Microenvironment Terms Signature Robustly Predicts the Prognosis and Immunotherapy Response in Bladder Cancer Based on Large Population Cohorts

**DOI:** 10.3389/fgene.2022.872441

**Published:** 2022-05-09

**Authors:** Shengjie Liang, Kai Fang, Simin Li, Dong Liu, Qingtong Yi

**Affiliations:** Department of Urology, Shanghai Pudong Hospital, Fudan University Pudong Medical Center, Shanghai, China

**Keywords:** bladder cancer, immune, signature, immunotherapy, prognosis

## Abstract

Immune microenvironment is implicated in cancer progression. However, the role of immune microenvironment in bladder cancer has not been fully explored. Open-accessed datasets GSE120736, GSE128959, GSE13507, GSE31684, GSE32548, GSE48075, GSE83586, and The Cancer Genome Atlas (TCGA) database were enrolled in our study. Single-sample gene set enrichment analysis (ssGSEA) was used to quantify 53 immune terms in combined BLCA cohorts. The top 10 important immune terms were identified through random forest algorithm for model establishment. Our model showed satisfactory efficacy in prognosis prediction. Furthermore, we explored clinical and genomic feature differences between high- and low-risk groups. The results indicated that the patients in the high-risk group might be associated with worse clinical features. Gene set enrichment analysis showed that epithelial–mesenchymal translational, mTORC1 signaling, mitotic spindle, glycolysis, E2F target, and G2M checkpoint pathways were aberrantly activated in high-risk patients, partially explaining its worse prognosis. Patients in the low-risk group showed better immunotherapy response according to TIDE and TCIA analysis, indicating that our model could effectively predict the immunotherapy response rate. KCNH4, UGT1A1, TPO, SHANK1, PITX3, MYH1, MYH13, KRT3, DEC1, and OBP2A genes were identified as feature genes in the high- and low-risk patients. CMAP analysis was performed to identify potential compounds targeting the riskscore.

## Introduction

Bladder cancer is one of the top 10 most common cancer types in the world, with approximately 570,000 new cases and 212,000 cancer-related deaths in 2020 (Sung et al., 2021). Of all cases, 90% of bladder cancer cases are bladder urothelial carcinoma (BLCA). Based on the degree of muscle invasion, bladder cancer can be categorized into two categories, non-muscle invasive bladder cancer (NMIBC, 75%) and muscle-invasive bladder cancer (MIBC, 25%). At present, surgical therapy is still the first-line choice for localized bladder cancers, although it has high postoperative recurrence and progression rates (Ghandour et al., 2019). For MIBC patients, poor prognosis and high metastasis rate are intractable problems in the clinic (Patel V. G. et al., 2020). Meanwhile, even for NMIBC patients, disease progression still occurs if not diagnosed and treated promptly. Given these limitations, it is essential to explore the molecular mechanism of bladder cancer progression and novel targets to predict the prognosis.

In the tumor microenvironment (TME), cancer cells can functionally reprogram the surrounding cells through the secretion of various cytokines, chemokines, and other factors (Hinshaw and Shevde, 2019). This recruitment effect could shift the local immune status and cell components in TME, contributing to tumor progression (Hinshaw and Shevde, 2019). For example, Cardoso et al. (2014) reported that human primary macrophages could induce Akt, c-Src, and ERK1/2 phosphorylation in gastric and colorectal cancer cells, thus leading to increased RhoA and Cdc42 activity. This axis stimulates gastric and colorectal cancer invasion. In breast cancer, Wang et al. (2018) found that tumor-associated macrophages facilitated breast cancer progression through activating NF-κB/SOX4 signaling in a CXCL1-dependent manner. In addition, the level of immune-related genes in tumor tissue is associated with cancer outcomes. Nakanishi et al. (2007) found that tumors with a higher level of PD-L1 were more likely to be considered high WHO grade. De Palma et al. (2008) reported that the immune treatment with IFN-α has yielded favorable outcomes in patients with melanoma. IFN-α promotes inflammatory environment, stimulates macrophages toward an M1 type, and hampers tumor growth and metastasis. Fiszer-Maliszewska et al. (1998) indicated that IL-2 could regulate adaptive immune response and increase infiltration of CD8^+^ T cells, further inhibiting tumor growth.

With the advancement of high-throughput sequencing technology, massive human genomic data have been accumulated, which brought great convenience for researchers. In this study, we comprehensively retrieved The Cancer Genome Atlas (TCGA) and Gene Expression Omnibus (GEO) databases and finally identified eight independent cohorts (1,614 samples) for further analysis. A total of 53 immune terms were quantified using the ssGSEA algorithm. Next, an immune term–based model was established based on the random forest algorithm. This model could effectively predict patients’ prognosis and associate with the BLCA immunotherapy response rate. Furthermore, KCNH4, UGT1A1, TPO, SHANK1, PITX3, MYH1, MYH13, KRT3, DEC1, and OBP2A genes in high- and low-risk groups showed a regular specific expression pattern. Moreover, Connectivity Map (CMAP) analysis was conducted to identify potential compounds targeting the riskscore used for BLCA treatment.

## Methods

### Data Acquisition

Transcription profiles and clinical information of BLCA patients stored in TCGA were downloaded from the TCGA-GDC website (https://portal.gdc.cancer.gov/). The reference file “Homo_sapiens.GRCh38.gtf obtained from Ensembl website (http://asia.ensembl.org/index.html) was used for gene annotation. GEO database (https://www.ncbi.nlm.nih.gov/gds/?term=) was comprehensively retrieved with the following terms: 1. searching terms were “(bladder) AND (cancer OR carcinoma)”; 2. the entry type was “series”; 3. the organism was “*Homo sapiens*”; and 4. the expression profile array was sequenced in tissues and the number of samples was larger than 50. Finally, seven individual GEO datasets were identified, including GSE120736 (platform: GPL10558), GSE128959 (platform: GPL6244), GSE13507 (platform: GPL6102), GSE31684 (platform: GPL570), GSE32548 (platform: GPL6947), GSE48075 (platform: GPL6947), and GSE83586 (platform: GPL16570). TCGA sequence data with FPKM form were converted into TPM form to better match microarray data and the code was obtained from https://haroldpimentel.wordpress.com/2014/05/08/what-the-fpkm-a-review-rna-seq-expression-units/. Sva package in R environment was used to perform data combining and magnitude harmonization, which resulted in a range between 1 and 20 of the expression value of each cohort (Leek et al., 2012). In detail, the ComBat function in sva package was used to remove batch effects and improve reproducibility. IMvigor210 cohort, a urothelial carcinoma cohort treated with the anti-PD-L1 antibody atezolizumab, was used for the prediction of patient response to immunotherapy, which was downloaded under Creative Commons 3.0 license. Differentially expressed genes (DEGs) were identified using limma package with a threshold of |logFC| > 1 and adj.*p* < 0.05 (Ritchie et al., 2015).

### Gene Set Enrichment Analysis and Single-Sample Gene Set Enrichment Analysis

The ClusterProfiler package was used to conduct gene set enrichment analysis (GSEA), which provided a gene classification method for pathway enrichment (Yu et al., 2012). The Hallmark gene set (MSigDB) was selected as a reference set to explore the difference in the oncogenetic pathways. Single sample GSEA (ssGSEA) was performed using the package GSVA in an R environment to evaluate the enrichment scores of 53 immune terms. The gene set used to quantify 53 immune terms has been uploaded in Figshare (https://figshare.com/s/d94792fd1f413eb68ca3) (Ren et al., 2021).

### Calculation of Immune Term–Based Riskscore in Combined BLCA Cohort

In the combined cohort that has been quantified into 53 immune terms, univariate Cox analysis was first performed to identify prognosis-related terms with the threshold of *p*-value <0.05. Random forest algorithm was then used for dimension reduction (ntree = 1,000), which was performed in R software using the randomForestSRC package (Alaa et al., 2019). The top 10 important terms were then identified for multivariate cox analysis and IBS calculation using the following formula: riskscore = coef1*term1 + coef2*term2 + coef3*term3 + … + coefN*termN. The patients were divided into high- and low-risk groups based on the median riskscore. ROC curve and Kaplan–Meier survival curve were used to evaluate the prognosis value of riskscore.

### Tumor Mutation and Immunotherapy Score

TCGA-BLCA tumor mutation data were downloaded from the cBioPortal website (http://www.cbioportal.org/datasets - Bladder Urothelial Carcinoma, TCGA, PanCancer Atlas). R software was used to take out the same sample as the expression data, including “Tumor_Sample_Barcode”, “Hugo_Symbol”, “Chromosome”, “Start_Position”, “End_Position”, “Variant_Classification”, “Variant_Type”, “Reference_Allele”, “Tumor_Seq_Allele1”, and “Tumor_Seq_Allele2”. The mutation types were distinguished into nonsynonymous and synonymous mutations. The tumor immune dysfunction and exclusion (TIDE) score was calculated based on the gene expression data, which could partly predict the immunotherapy response rate of enrolled patients (http://tide.dfci.harvard.edu/login/) (Fu et al., 2020). In addition, the result of comprehensive immunogenomic analyses was obtained from The Cancer Immunome Database (TCIA) website (https://tcia.at/home).

### Connectivity Map Analysis

The connectivity map (CMAP) is a gene expression profile database based on intervention gene expression developed by the Broad Institute. It is mainly used to reveal the functional relationship between small molecular compounds, genes, and disease status. The top 500 upregulated and downregulated genes between high- and low-risk patients were identified as input files of CMAP analysis. The small bioactive molecules that were potential targets for BLCA immune signature were then screened based on these DEGs. ComplexHeatmap package was used for result visualization (Gu et al., 2016).

### Statistical Analysis

All the analyses were performed using R software (R Core Team, version 4.0.0) and GraphPad Prism version 7.0. All statistical tests were two-sided, and *p*-value <0.05 was considered statistically significant. Continuous variables with normal distribution in two groups were compared using an independent *t*-test. Continuous variables with skewed distribution in two groups were compared with Wilcoxon rank-sum test. Kruskal–Wallis test was used to compare the data in more than two groups. The log-rank test was used to compare the statistical difference in the Kaplan–Meier survival curve. The ggplot2 package was used to perform most graphic drawings (https://ggplot2.tidyverse.org/). The R code and primary data in this study were uploaded in Figshare (https://figshare.com/s/d94792fd1f413eb68ca3).

## Results

### Data Combination and Immune Term Quantification

**FIGURE 1:**
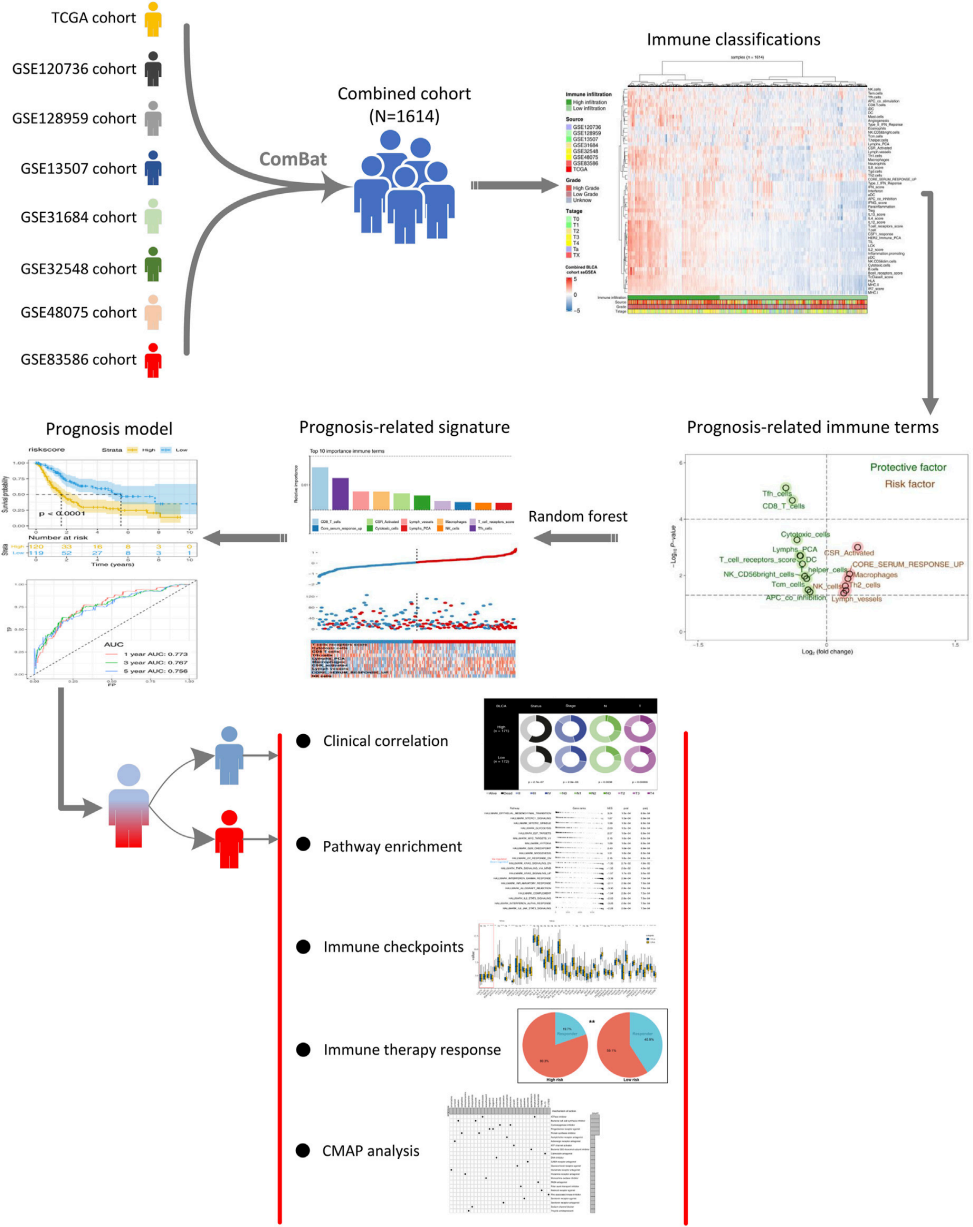
Whole flowchart of our analysis. Notes**:** Eight independent BLCA cohorts were screened and combined into a large cohort for further analysis using sva package. ssGSEA package was used to quantify 53 immune terms. The top 10 important immune terms were identified through random forest algorithm for model establishment. The model showed satisfactory efficacy in prognosis prediction. We further explored the clinical and genomic differences between high- and low-risk patients.

**FIGURE 2:**
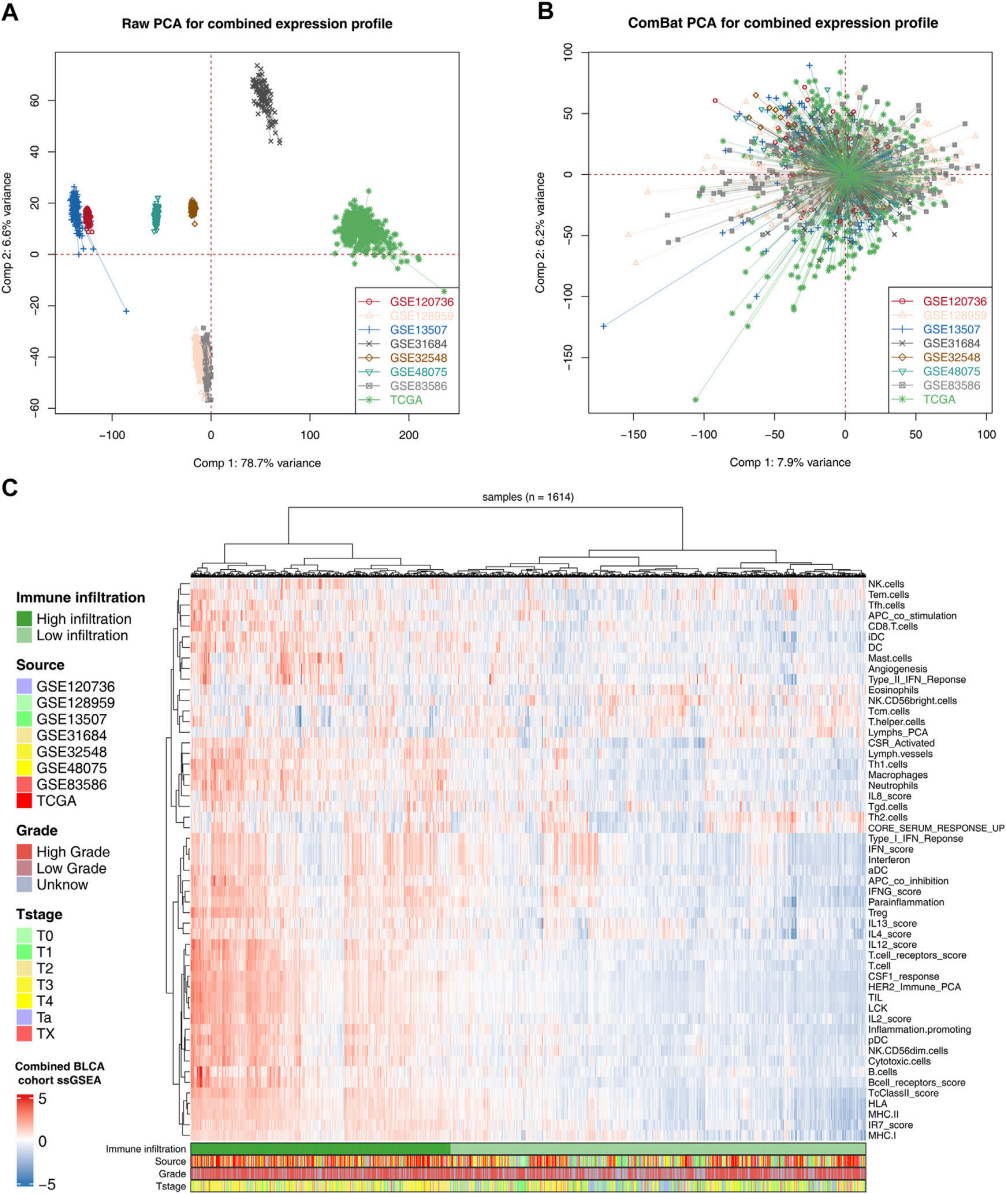
Combination of BLCA cohort and quantification of immune terms. Notes: **(A)** Eight BLCA cohorts selected for our analysis have noticeable batch differences; **(B)** sva package used for BLCA cohort combination greatly reduces the batch difference; **(C)** expression profile of all patients was quantified as 53 immune terms using ssGSEA package.

The flowchart of the whole study is shown in Figure 1. After comprehensively retrieving the GEO and TCGA database, eight BLCA cohorts met our criteria, including GSE120736, GSE128959, GSE13507, GSE31684, GSE32548, GSE48075, GSE83586, and TCGA database (Figure 2A). The detailed clinicopathological characteristics of patients in the TCGA cohort and each GEO cohort have been summarized in Supplementary Table S1–8. Meanwhile, we found a lot of inter-assay variances in these datasets (Figure 2A; Comp 1: 78.7% variance, Comp 2: 6.6% variance). Therefore, we used sva package to eliminate potential inter-assay differences as much as possible and to generate a combined BLCA cohort (Figure 2B; 1,614 samples). Furthermore, we used the ssGSEA package to quantify patients’ gene expression profiles into 53 immune terms for further analysis (Figure 2C).

### Immune Term-Based Prognosis Model

**FIGURE 3:**
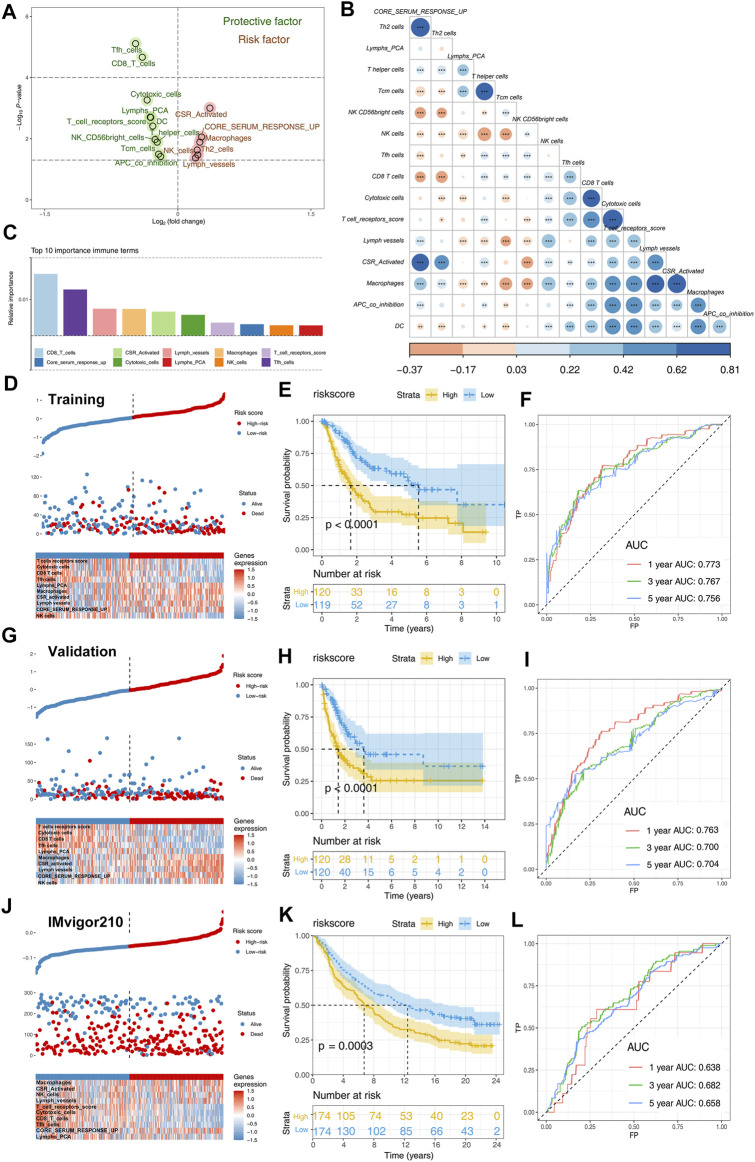
Identification of prognosis-related immune terms and riskscore calculation. Notes: **(A)** Univariate Cox analysis identified prognosis-related terms, including 10 protective and six risk factors; **(B)** co-expression relationship of these 16 prognosis-related terms; **(C)** top 10 important terms identified by random forest algorithm; **(D)** risk plot of patients in training cohort; **(E)** Kaplan–Meier showed that high-risk patients tend to have a worse prognosis in training group; **(F)** ROC curves indicated a satisfactory prediction efficacy of the model in the training group; **(G)** risk plot of patients in validation cohort; **(H)** Kaplan–Meier showed that high-risk patients tend to have a worse prognosis in the validation group; **(I)** ROC curves indicated a satisfactory prediction efficacy of the model in the validation group; **(J)** risk plot of patients in IMvigor210 cohort; **(K)** Kaplan–Meier showed that high-risk patients tend to have a worse prognosis in the IMvigor210 group; **(L)** ROC curves indicated a satisfactory prediction efficacy of the model in the IMvigor210 group.

Patients with complete survival information, including survival time and status, were selected for further analysis to explore the effect of identified immune terms on patients’ prognosis. First, the patients in combined cohorts with complete prognostic information were randomly divided into training and validation groups at a ratio of 1:1. Then, we performed univariate Cox analysis to identify prognosis-related terms. The result showed that 10 terms were protective factors, and six were risk factors for the BLCA patients (Figure 3A). Co-expression relationships of these prognosis-related terms are shown in Figure 3B. Next, we performed a random forest algorithm to calculate the relative importance of these prognosis-related terms (Supplementary Figure S1, oob error rate = 0.373 when ntree = 1,000). The top 10 important terms (CD8_T_cells, core serum response up, CSR_activated, cytotoxic cells, lymph vessels, lymphs_PCA, macrophages, NK cells, T_cell_receptors_score, and Tfh cells) were selected for further multivariate cox analyses (Figure 3C). Then, a prognosis model based on these immune terms was established with the formula of “riskscore = CD8_T_cells * -0.1168 + Tfh cells * -0.1346 + lymph vessels * -0.0163 + macrophages * 0.3199 + CSR_activated * 0.0280 + cytotoxic cells * -0.2001 + T_cell_receptors score * -0.1205 + core serum response up * 0.1471 + NK cells * 0.0745 + Lymphs_PCA * -0.1213. The risk plot of patients in the training cohort is shown in Figure 3D. The Kaplan–Meier survival curve indicated that the patients in the high-risk group might have worse overall survival than patients in the low-risk group (Figure 3E; *p* < 0.0001). In parallel, the ROC curve showed a satisfactory predicted efficacy on patients’ prognosis in the training group (Figure 3F; 1-year AUC = 0.773, 3-year AUC = 0.767, and 5-year AUC = 0.756). Also, our prognosis model showed an effectively predicted efficacy in the internal validation group (Figures 3G–I; 1-year AUC = 0.763, 3-year AUC = 0.700, and 5-year AUC = 0.704) and IMvigor210 external cohort (Figure 3J–L; 1-year AUC = 0.638, 3-year AUC = 0.682, and 5-year AUC = 0.658). Univariate and multivariate Cox regression analysis showed that our model has satisfactory prognostic efficiency independent of clinical factors (Supplementary Figure S2).

### Clinical and Biological Relevance of Riskscore

**FIGURE 4:**
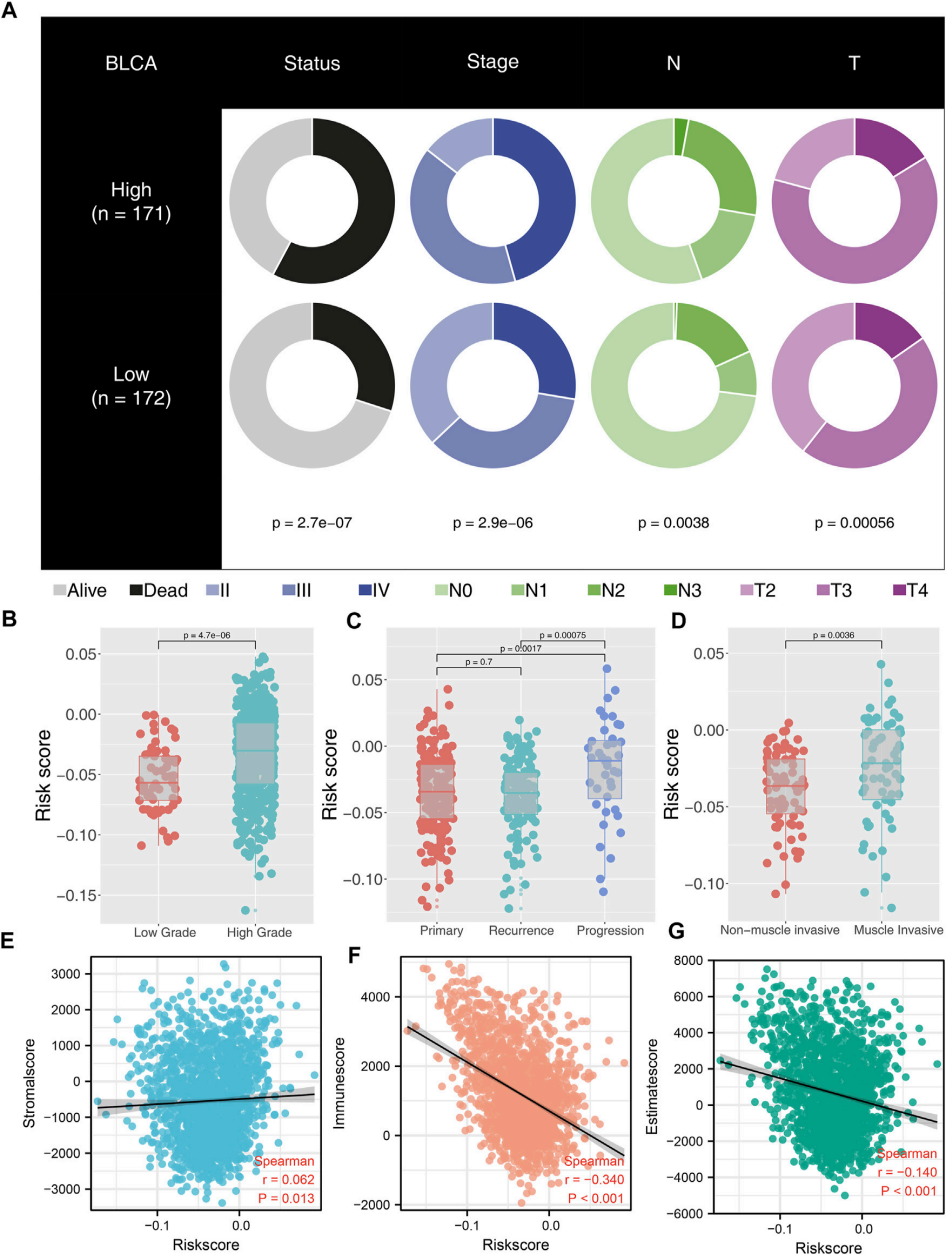
Correlation between riskscore and clinical features of BLCA patients. Notes: **(A)** high-risk patients have worse clinical features; **(B)** high-grade patients have higher riskscore than low-grade patients; **(C)** patients with disease progression have higher riskscore than primary and recurrence patients; **(D)** muscle invasive patients have higher riskscore than non-muscle invasive patients; **(E)** correlation between riskscore and stromalscore; **(F)** correlation between riskscore and immunescore; **(G)** correlation between riskscore and estimatescore.

Clinical and biological relevance were explored to probe into the possible mechanism of our model on patients’ prognosis. We found that high-risk patients might have adverse baseline characteristics (Figure 4A). Moreover, patients with high grade tend to have higher riskscore than patients with low grade (Figure 4B). Interestingly, we found that riskscore was not associated with disease relapse but with disease progression (Figure 4C). In addition, we explored the riskscore difference between non-muscle invasive and muscle invasive BLCA and found that muscle invasive patients had higher riskscore values (Figure 4D). Estimate package quantified tumor microenvironment into immunescore, stromalscore, and estimatescore, standing for the content of immune, stromal, and non-tumor cells, respectively (Figures 4E–G). Our result showed that riskscore was significantly negatively correlated with immunescore and estimatescore (Figures 4F,G; immunescore, r = -0.340, *p* < 0.001; estimatescore, r = -0.140, *p* < 0.001).

### Pathway Enrichment and Tumor Mutation Burden

**FIGURE 5:**
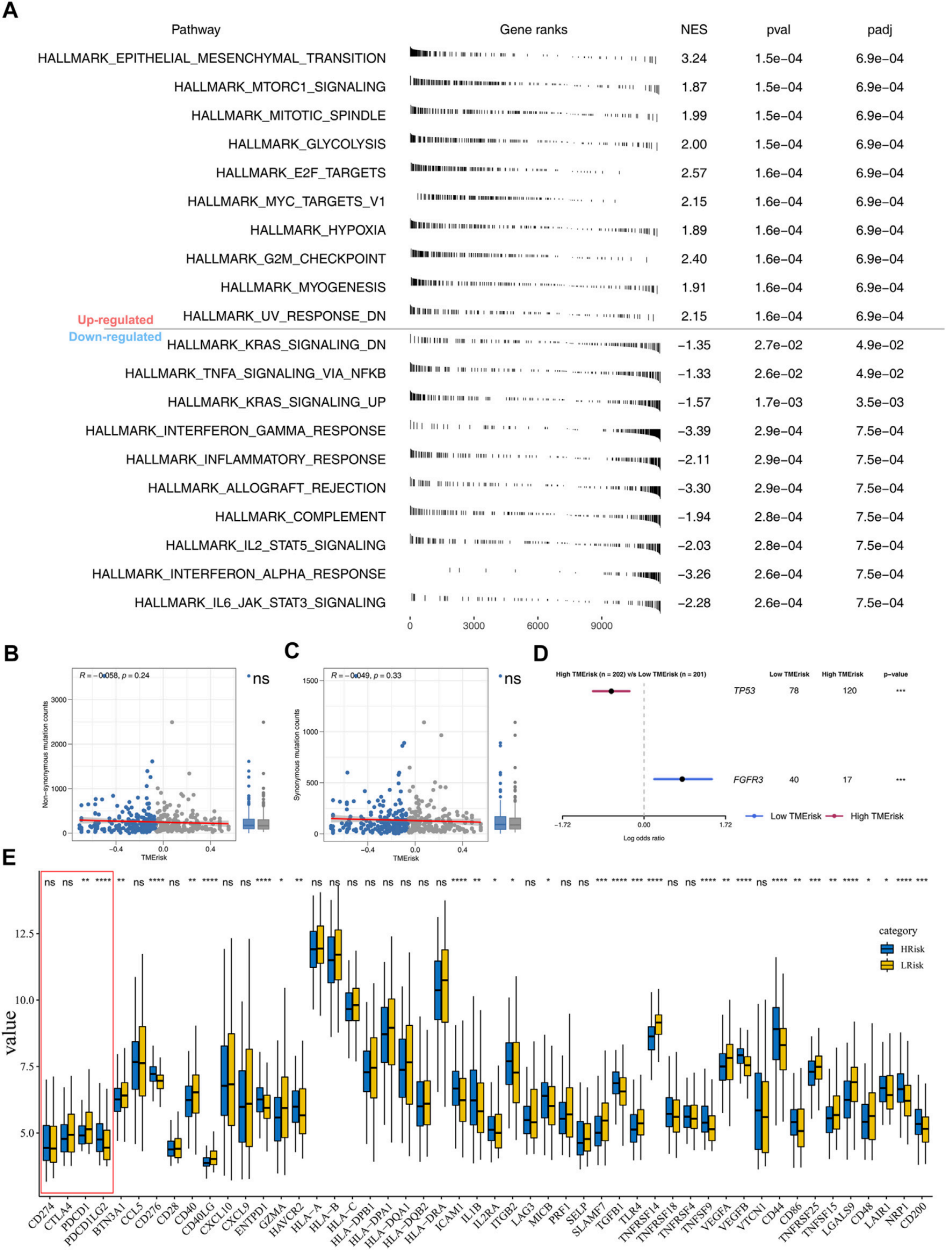
Pathway enrichment analysis and tumor burden. Notes: **(A)** GSEA was performed to explore biological pathway difference between high- and low-risk patients; **(B–C)** non-synonymous mutation and synonymous mutation between high- and low-risk patients; **(D)** percentage of TP53 and FGFR3 mutation was significantly different in high- and low-risk patients; **(E)** immune gene expression in high- and low-risk groups.

With the Hallmark gene set as a reference set, we performed GSEA to explore the underlying oncogenetic difference between high- and low-risk patients. The result showed that the epithelial–mesenchymal translational (EMT), mTORC1 signaling, mitotic spindle, glycolysis, E2F target, and G2M checkpoint pathways were aberrantly activated in high-risk patients (Figure 5A). No remarkable difference was found in non-synonymous mutation and synonymous mutation between high- and low-risk patients (Figures 5B,C). However, we observed more TP53 mutation in high-risk patients (low-risk: 78; high-risk: 120) and FGFR3 mutation in low-risk patients (low-risk: 40; high-risk: 17) (Figure 5D).

### Riskscore was Associated With Patients’ Immunotherapy Response Rate

**FIGURE 6:**
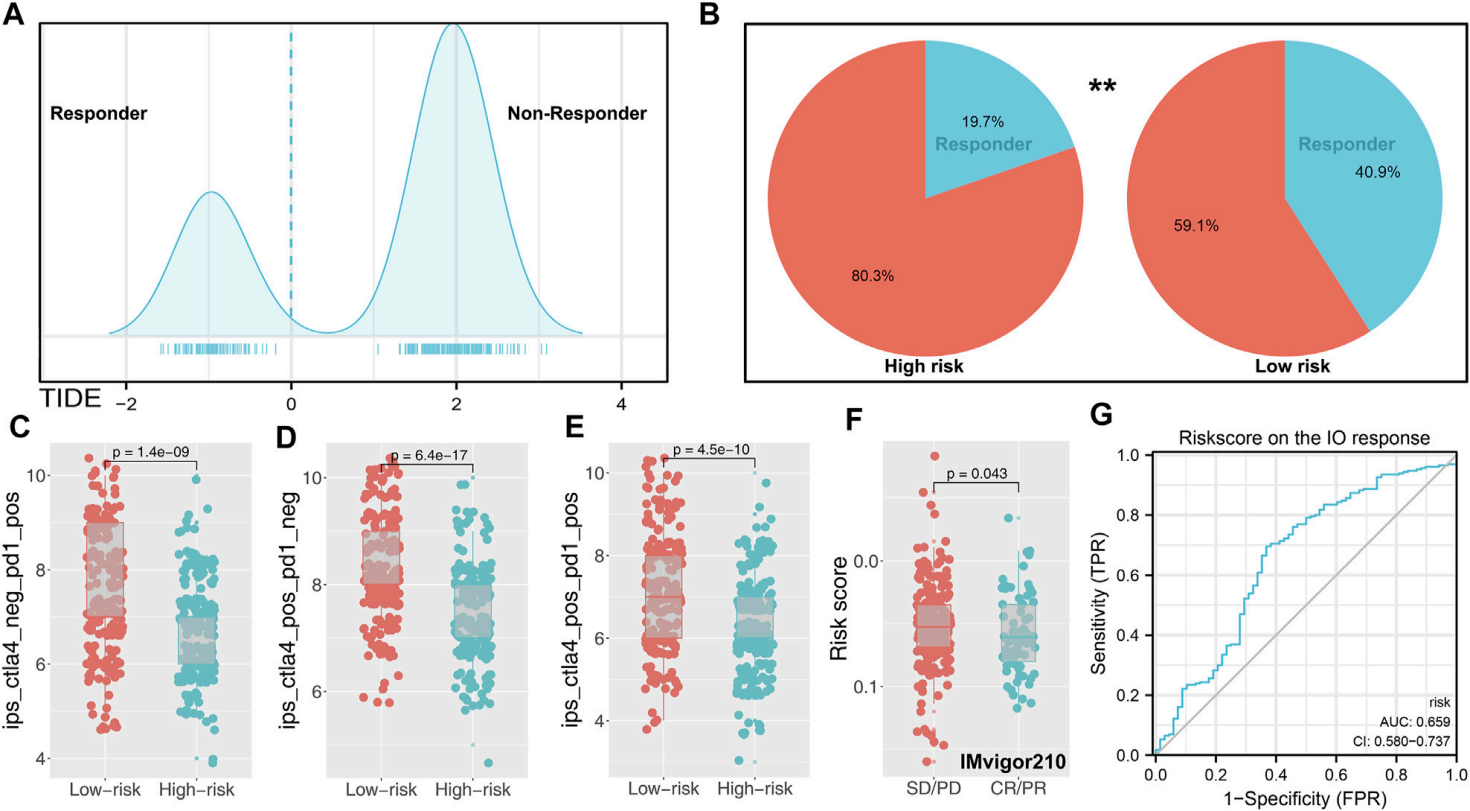
Immune term-based model was associated with immunotherapy response. Notes: **(A)** TIDE score of each patients was obtained from http://tide.dfci.harvard.edu/(TIDE score >0 was defined as nonresponder); **(B)** low-risk group has a higher percentage of responders; **(C–E)** difference of TCIA immunotherapy score between high- and low-risk patients; **(F)** risksocre was lower in immunotherapy complete/partial response patients (IMvigor210 cohort); **(G)** ROC curve indicated the prediction efficiency of riskscore on the immunotherapy response in IMvigor210 patients.

Nowadays, immunotherapy has become an important therapeutic strategy for BLCA patients. Therefore, we assessed riskscore differences in multiple checkpoint molecules (Figure 5E). To be noted, we found statistically significant differences of critical immune checkpoint molecules PD-L1 and PD-L2 between high- and low-risk patients, indicating that the immunotherapy response rate might differ in these two groups (Figure 5E). Furthermore, we performed TIDE analysis of our combined cohort (Figure 6A). TIDE score >0 was defined as nonresponders, while they were defined as responders otherwise (Figure 6A). More responders were observed in the low-risk group than in the high-risk group (Figure 6B). TCIA results also reached the same conclusion. The result demonstrated that the low-risk group had higher TCIA scores (CTLA4_negative_PD1_positive, CTLA4_positive_PD1_negative, CTLA4_positive_PD1_positive) than the high-risk group, indicating that low-risk might have a better immunotherapy efficacy of anti-CTLA4 and anti-PD-1 therapy (Figures 6C–E). Meanwhile, we validated this conclusion in a urothelial carcinoma cohort treated with the anti-PD-L1 antibody atezolizumab, IMvigor210. The result showed that riskscore was lower in immunotherapy complete/partial response patients (Figure 6F). Moreover, the ROC curve showed that the riskscore could effectively predict the immunotherapy response of patients in IMvigor210 (Figure 6G, AUC = 0.659; SD/PD and CR/PR).

### Identification of Marker Gene and Candidate Compounds Targeting the Riskscore

**FIGURE 7:**
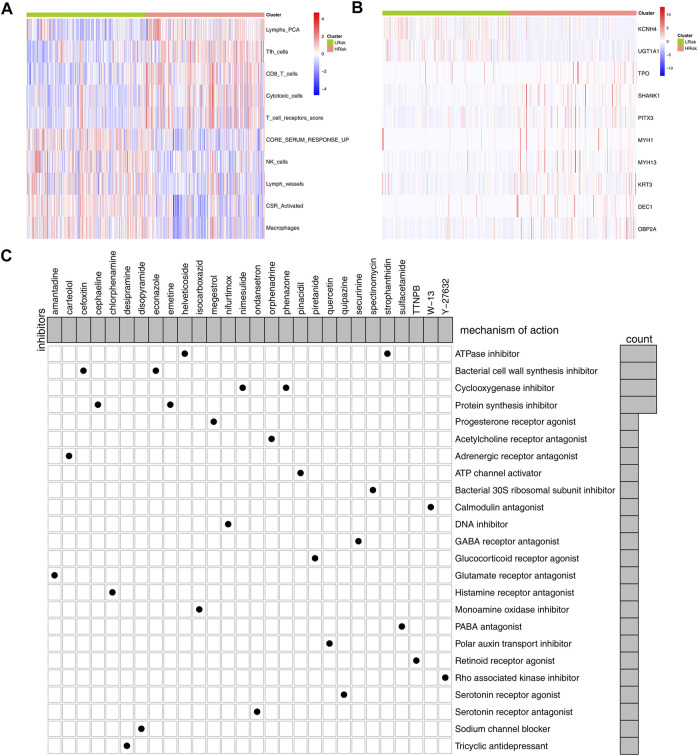
Identification of marker genes and candidate compounds. Notes: **(A,B)** differential immune infiltrating patterns and genes between the high- and low-risk group were illustrated in heatmap; **(C)**: CMAP analysis was performed to identify potential compounds targeting the riskscore.

Our model could effectively indicate patients’ prognosis and immunotherapy response rate. Specific marker genes contributed to the quick genotyping in clinical features. For the model immune terms, Lymphs_PCA, Tfh cells, CD8_T_cells, cytotoxic cells, and T_cell_receptors score was obviously activated in high-risk patients, whereas core serum response up, NK cells, lymph vessels, CSR_activated, and macrophages were downregulated (Figure 7A). At the same time, 10 characteristic genes were identified between high- and low-risk patients, including KCNH4, UGT1A1, TPO, SHANK1, PITX3, MYH1, MYH13, KRT3, DEC1, and OBP2A (Figure 7B). The clinical correlation and KM survival curves of these feature genes are shown in Supplementary Figure S3. The result showed that TPO, SHANK1, PITX3, MYH13, KRT3, and DEC1 were associated with worse clinical features and prognosis, yet the KCNH4 and UGT1A1 were the contrary. Based on the logistic regression, the expression level of these 10 genes could effectively predict the risk group of bladder cancer patients (Supplementary Figure S4A, AUC = 0.856). Meanwhile, these 10 genes also have some predictive power on the immunotherapy response rate in the IMvigor210 cohort (Supplementary Figure S4B, AUC = 0.620). Based on the CMAP analysis, potential compounds targeting the riskscore were screened, for example, amantadine, carteolol, and helveticoside (Figure 7C). The mechanism of these compounds was mainly focused on ATPase inhibitor, bacterial cell wall synthesis inhibitor, cyclooxygenase inhibitor, progesterone receptor agonist, and protein synthesis inhibitor.

## Discussion

Globally, as the most common urinary tract malignancy, bladder cancer is still a leading cause of mortality (Dobruch et al., 2016). Immunotherapy has been explored in bladder cancer with encouraging results, making it an important treatment choice for advanced bladder cancer (Lenis et al., 2020). This study developed a new scoring tool that can effectively indicate bladder cancer patients’ survival and immunotherapy response.

First, eight BLCA cohorts were combined using sva package to reduce bias brought by the small sample size and then quantified into 53 immune terms. Univariate Cox analysis identified 16 immune terms closely associated with patients’ prognosis (10 protective and six risk factors). The top 10 important terms, CD8_T_cells, core serum response up, CSR_activated, cytotoxic cells, lymph vessels, lymphs_PCA, macrophages, NK cells, T_cell_receptors_score, and Tfh cells, were selected by random forest algorithm for model construction, indicating their vital role in BLCA progression. Liu et al. (2020) demonstrated that high infiltration of TIGIT + CD8^+^ T cells was associated with worse overall and recurrence-free survival in BLCA. Mukherjee et al. (2018) revealed that intratumoral CD56 ^+^ NK cells were frequent in lymphoid tissues and associated with better prognosis, while CD56^−^ NK cells were the opposite. Meanwhile, a systemic review conducted by Wu et al. (2019) indicated that lymphangiogenesis and lymph node invasion might influence cancer cell spread. Also, lymph vessels could affect tumor immunity (for instance, transporting antitumor CD8^+^ T cells) to hamper localized tumor tissues playing a dual role in the tumor environment. Martínez et al. (2017) found that BMP4 could induce monocyte/macrophage toward M2 phenotype macrophages, favoring bladder cancer progression. With the deepening of research on tumor immunity, the vital immune terms identified in our study might provide a basis for further exploration of the immune microenvironment of bladder cancer.

We further explored the underlying biological difference between high- and low-risk patients through GSEA and burden analysis. GSEA results showed that the EMT, mTORC1 signaling, mitotic spindle, glycolysis, E2F target, and G2M checkpoint pathways were significantly upregulated in the high-risk group. EMT is a process in which epithelial cells transform into motile mesenchymal cells, which is related to malignant biological behavior (Lamouille et al., 2014). Multiple studies have been reported that EMT could accelerate cancer cell proliferation and invasion (Xie et al., 2020; Zhang et al., 2020). Meanwhile, the mTOR signaling pathway was widely involved in bladder cancer development. Patel R. et al. (2020) found that the concurrent suppression of atypical protein kinase-C (PKC) and mTOR could remarkably inhibit bladder cancer progression. The mitotic spindle is a fundamental physiological process in cells. The abnormal mitotic spindle in cancer cells might increase cellular heterogeneity, facilitating genomic instability, metastasis, and cancer stemness (Pease and Tirnauer, 2011). G2/M checkpoint is a limitation step of the cell cycle and its arrest could inhibit cell proliferation (Akaike and Chibazakura, 2020). These findings indicated that the patients with high-risk might aberrantly activate the aforementioned pathway, thus leading to worse genomic and prognosis characteristics.

Furthermore, we found higher PD-L1 and PD-L2 levels in low-risk patients. The expression of PD-L1 has been reported as a predictive biomarker in cancer immunotherapy (Patel and Kurzrock, 2015). Meanwhile, some anti-PD-1/PD-L1 drugs such as atezolizumab have achieved promising results in bladder cancer therapy (Inman et al., 2017). TIDE and TCIA analysis showed that low-risk patients have a better immunotherapy response rate than high-risk patients, which might be associated with a higher PD-L1 level in low-risk patients. Meanwhile, we observed an increased FGFR3 mutation percentage in the low-risk group. From previous studies, FGFR3 is a prognosis marker and could affect the immunotherapy response rate of bladder cancer (Kacew and Sweis, 2020). Moreover, Wang et al. (2019) found that the bladder cancer patients with FGFR3 mutation might be the suboptimal candidates for PD-1/PD-L1 inhibitor. CMAP analysis identified multiple compounds targeting the riskscore, including some ATPase inhibitors, bacterial cell wall synthesis inhibitors, cyclooxygenase inhibitors, progesterone receptor agonists, and protein synthesis inhibitors. This means that if the patient is determined to have high riskscore characteristics, the addition of these small molecular compounds to their treatment regimen might reduce their riskscore characteristics. In our study, high-risk patients tend to have a worse prognosis and higher genomic instability. The application of these molecular compounds and others with similar chemical structural formulas may improve patients’ OS and reduce genomic instability. Meanwhile, we identified 10 feature genes between the high- and low-risk group. The result showed that the expression level of these 10 genes could effectively predict the risk group of bladder cancer patients. Therefore, detecting the relative expression levels of these genes during cystoscopic biopsy could provide a certain reference value for clinical application.

There are several limitations to the study. First, the samples included in our analysis were predominantly from the Western populations, which might bring population and genomic bias. However, the Asian population cohort with large samples and complete clinical information remains scarce. Therefore, the inclusion of subsequent Asian population cohort meeting the criteria could improve the stability of our results. Second, not all the samples included in this study had complete prognosis information, including survival time and status. Third, there are no appropriate bladder cancer tissues and cell lines in CMAP analysis, that is, although the CMAP analysis is performed based on the genomic change, the corresponding result was trained in other cell lines, such as PC-3 and MCF-7, which might reduce the reproducibility of identified compounds. Fourth, TPM counts generated from RNA sequencing are not usually performed for urothelial cancer patients, which is the main resistance for the clinical application of our model. Despite these limitations, our study developed a robust signature for predicting the overall survival of BLCA patients and was validated in training and validation cohorts with large populations. In addition, our result indicated that the model was associated with patients’ immunotherapy response rate.

## Conclusion

Here, our study developed an immune term–based model based on the large BLCA cohort, which is a powerful tool for predicting the prognosis and immunotherapy response rate of BLCA patients. Patients in the high-risk group were associated with worse clinical and prognosis features. GSEA showed that multiple oncogenic pathways were upregulated in high-risk patients. The potential compounds targeting our model were identified. KCNH4, UGT1A1, TPO, SHANK1, PITX3, MYH1, MYH13, KRT3, DEC1, and OBP2A were identified as characteristic genes implicated in differences in prognosis and immunotherapy response rates in the high- and low-risk group.

## Data Availability

The original contributions presented in the study are included in the article/Supplementary Material, further inquiries can be directed to the corresponding author.
