# Supplementary figures and images for: Immune Microenvironment Terms Signature Robustly Predicts the Prognosis and Immunotherapy Response in Bladder Cancer Based on Large Population Cohorts
Source: Front Genet. 2022 May 9;13:872441. doi: 10.3389/fgene.2022.872441 (PMC9126043; doi:10.3389/fgene.2022.872441)

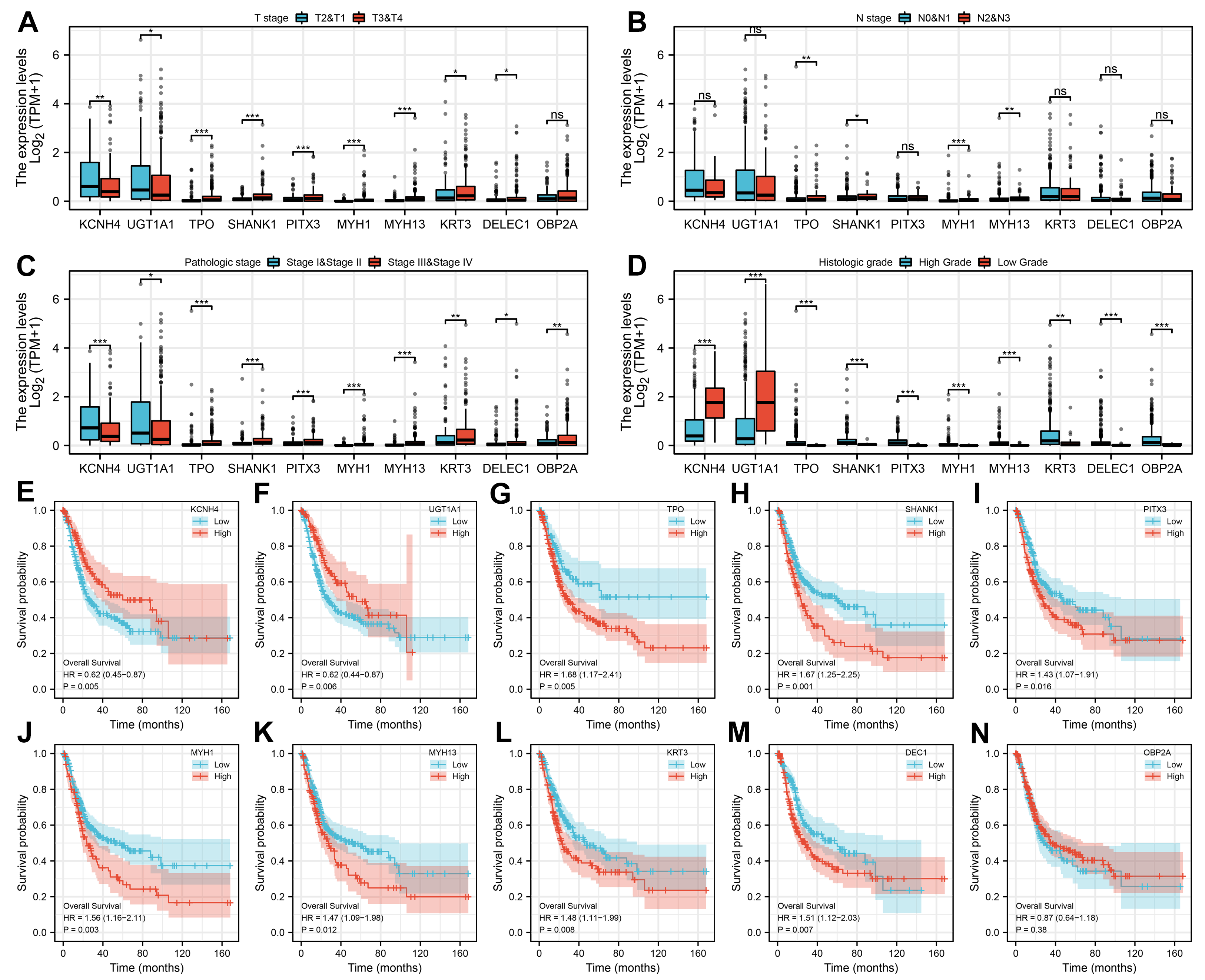

Supplement: Supplementary file 1 [file Image3.TIF]

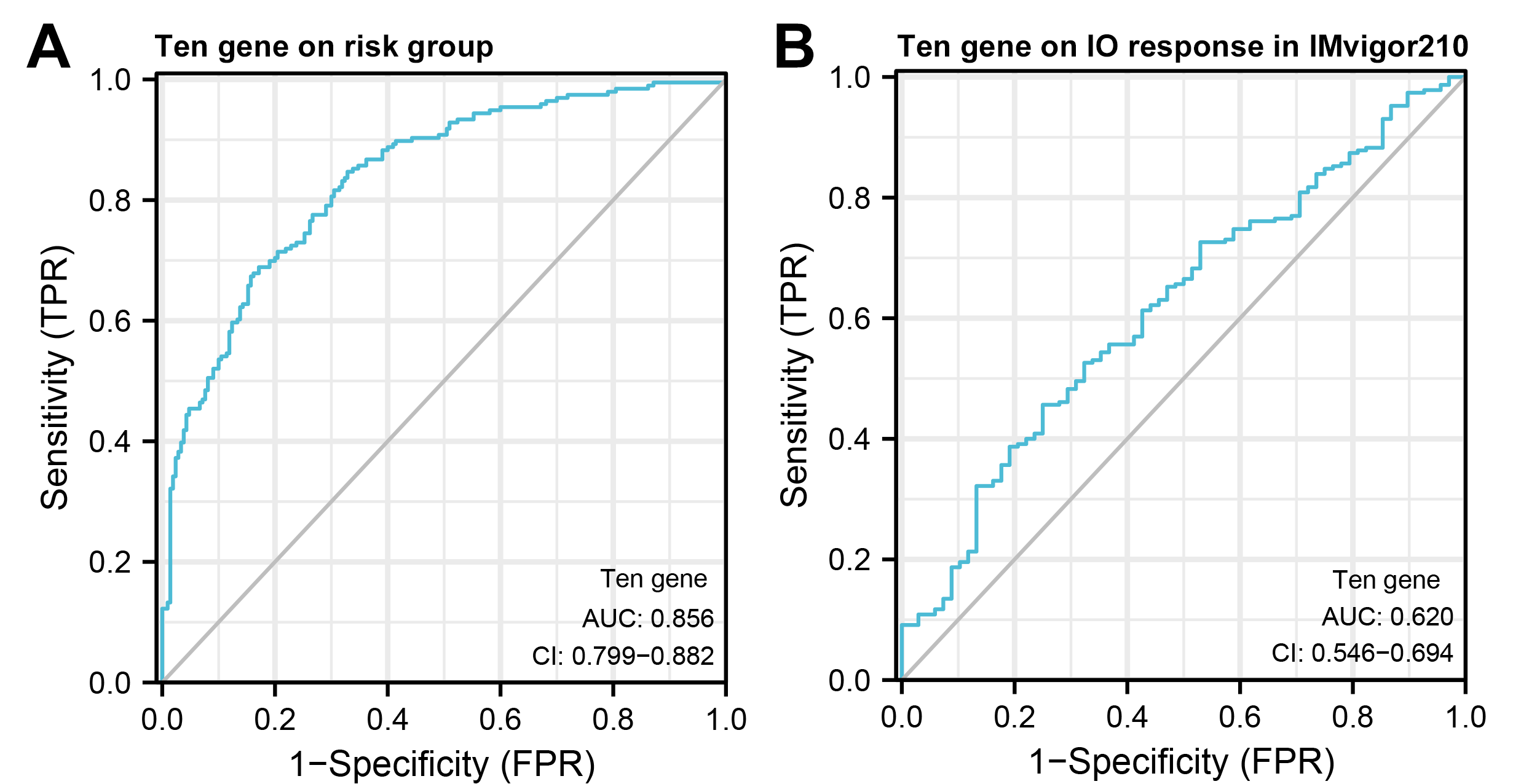

Supplement: Supplementary file 2 [file Image4.TIF]

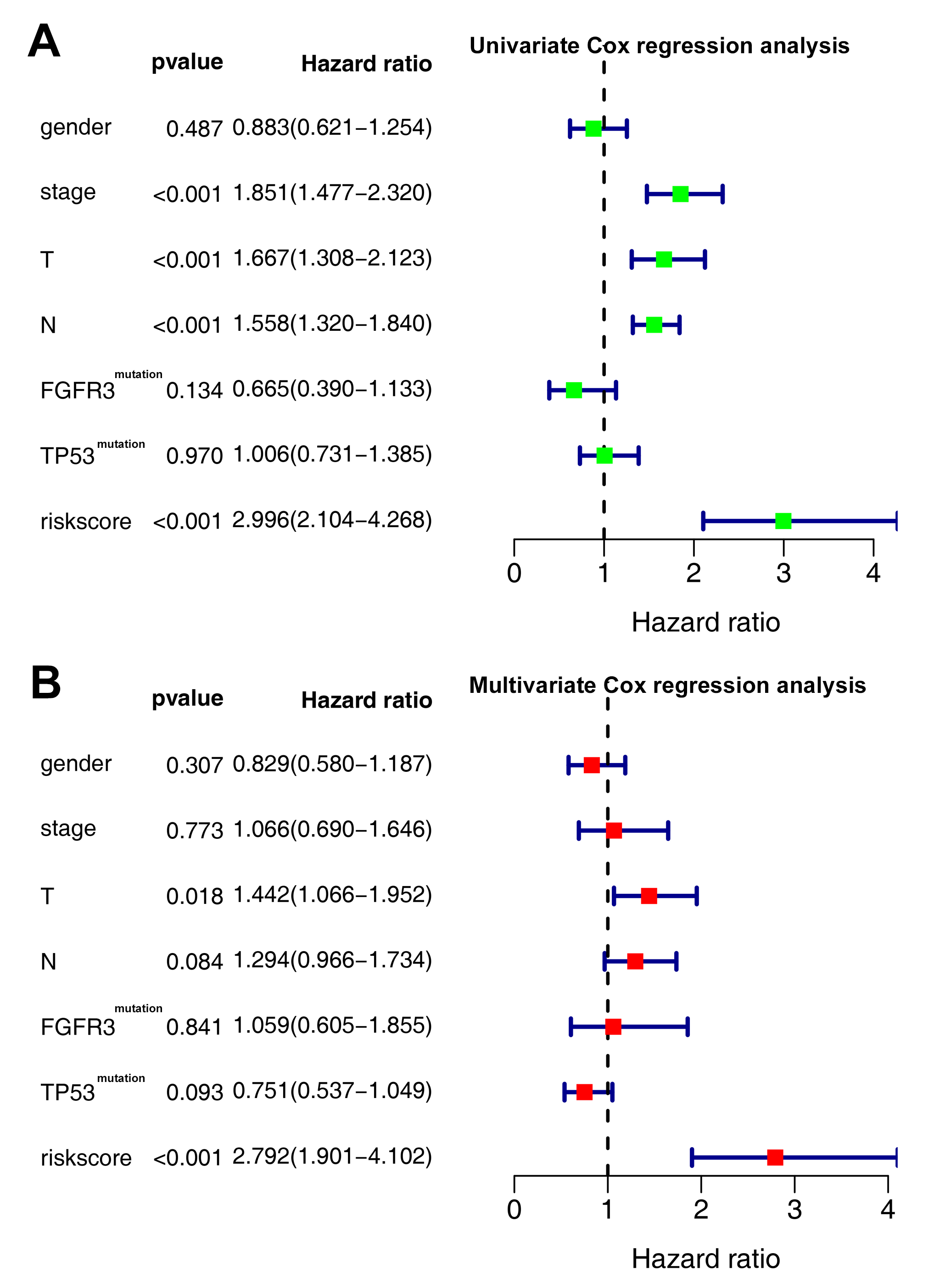

Supplement: Supplementary file 3 [file Image2.TIF]

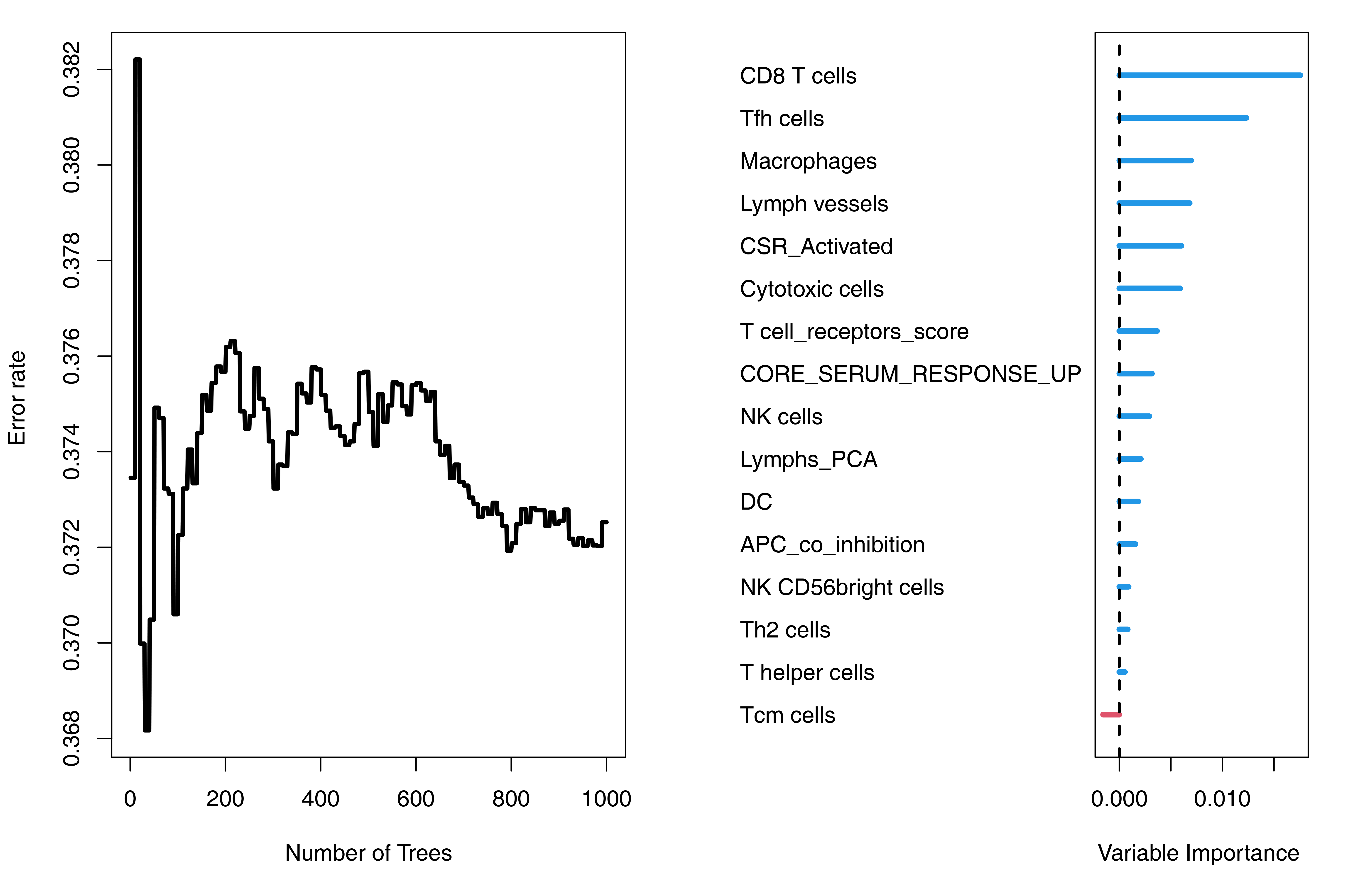

Supplement: Supplementary file 4 [file Image1.TIF]
